# IL-1R1 deficiency impairs liver regeneration after 2/3 partial hepatectomy in aged mice

**DOI:** 10.3906/biy-2010-51

**Published:** 2021-04-20

**Authors:** Deming LI, Ze WANG, Chunyan ZHANG, Cunshuan XU

**Affiliations:** 1 State Key Laboratory Cell Differentiation and Regulation, Henan Normal University, Xinxiang, Henan China; 2 Henan International Joint Laboratory of Pulmonary Fibrosis, Henan Normal University, Xinxiang, Henan China; 3 Henan Center for Outstanding Overseas Scientists of Pulmonary Fibrosis, Henan Normal University, Xinxiang, Henan China; 4 College of Life Science, Henan Normal University, Xinxiang, Henan China; 5 Institute of Biomedical Science, Henan Normal University, Xinxiang, Henan China; 6 Overseas Expertise Introduction Center for Discipline Innovation of Pulmonary Fibrosis (111 Project), Henan Normal University, Xinxiang, Henan China

**Keywords:** Aged mice, *IL-1R1*, inflammation, liver resection, regeneration

## Abstract

Inflammation has a dual effect: it can protect the body and destroy tissue and cell as well. The purpose of this experiment was to determine the role of *IL-1R1 *in liver regeneration (LR) after partial hepatectomy (PH) in aged mice. The wild-type (WT, n = 36) and the *IL-1R1* knockout (KO, n = 36) 24-month-old C57BL/6J mice underwent two-thirds PH; 33 WT mice underwent sham operation. Liver coefficient was calculated by liver/body weight. The mRNA and protein expressions of genes were evaluated by quantitative real-time polymerase chain reaction (qRT-PCR) and Western blotting methods, respectively. Compared with WT mice, liver coefficient was lower in the *IL-1R1* KO aged mice at 168 and 192 h (p = 0.039 and p = 0.027). The mRNA transcription of inflammation-related genes and cell cycle-associated genes decreased or delayed. The protein expressions of proliferation-related marker PCNA and proliferation-associated signaling pathway components JNK1, NF-κB and STAT3 reduced or retarded. There was stronger activation of proapoptotic proteins caspase-3, caspase-8 and BAX in the *IL-1R1 *KO mice at different time points (p < 0.05 or p < 0.01). *IL-1R1* KO reduced inflammation and caused impaired liver regeneration after 2/3 partial hepatectomy in aged mice. Maintaining proper inflammation may contribute to regeneration after liver partly surgical resection in the elderly.

## 1. Introduction

The liver has a strong regenerative ability. When it encounters with viral infections, toxins or partial hepatectomy (PH) injury, it can recover the original mass and functions by regeneration (Ibrahim and Weiss, 2019). The rodent two-thirds PH is one of the most effective models to study liver regeneration (LR). After removal of left and middle lobes, a series of cytokines, growth factors, hormones and signaling pathways are activated, which drive progression of hepatocytes through three distinct phases: priming, proliferation and termination phase; ultimately it restores to the original volume and the liver/body weight ratio (Fausto et al., 2006). Effective liver regeneration is of important clinical significance, which can decrease morbidity and mortality after serious liver trauma, cancer resection and donor liver transplantation (Sato et al., 2019).

With the extension of life expectancy, the number of elderly people is increasing. Aging alters the biological processes of many organs and tissues, resulting in the development of age-related diseases and abnormal body homeostasis. With the aging of the heart, lung, kidney and other organs, the changes of pathophysiology and the decrease of organ function occurred; the liver also changed with aging, but the liver function remained relatively stable (Iakova et al., 2003). 

IL-1 is a very important mediator of innate immune and inflammatory diseases, also known as proinflammatory cytokines. Many biological functions of IL-1 are mediated by interleukin-1 receptor (IL-1R). IL-1R has 10 family members; the main members are interleukin-1 receptor 1 (IL-1R1) and interleukin-1 receptor 2 (IL-1R2). Among IL-1 family members, 2 active molecules IL-1α and IL-1β and a receptor antagonist IL-1Ra, can be linked to IL-1R1. After IL-1α or IL-1β was connected with IL-1R1, IL-1R1 and the coreceptor IL-1RAcP form a heterodimer, which allows signal transduction molecules TNFR associated factor 6 (TRAF6) or myeloid differentiation protein 88 (MyD88) or IL-1R associated kinase 4 (IRAK4) to connect to the TIR domain of IL-1R1 and IL-1RAcP heterodimer (Boraschi and Tagliabue, 2013). IL-1α or IL-1β can also be connected to IL-1R2. IL-1R2 cannot induce intracellular signals, but only acts as a decoy receptor for IL-1α and IL-1β. IL-1R2 plays an inhibitory role in IL-1 activity and is a compensation for IL-1Ra function. IL-1Ra is competitively connected to the IL-1 receptor without activating the downstream channel, which is an endogenous inhibitor of IL-1α and IL-1β. The expression of balance among IL-1, IL-1Ra and IL-1R plays a decisive role in the establishment of proinflammatory and steady-state function (Gunther et al., 2017). In some physiological conditions, e.g., low expression level of brain IL-1 can enhance the organism’s ability to adapt to the stimulus, so as to promote its efficient response; but in some chronic or acute responses, repression of IL-1 expression may be used as an effective means of prevention and treatment (Goshen and Yirmiya, 2009). In a starvation experiment, *IL-1R1* knockout (KO) aged mice were found to be able to increase intestinal atrophy and reduce cell proliferation (Song et al., 2011). Feng et al. reported that IL-1R1 is required for antiobesity (Feng et al., 2019). IL-1/IL-1R1-signaling were found to have protection function in bacterial infection (Moorlag et al., 2020). However, recently study suggested IL-1R1 signaling had adverse effect on the onset of acute liver injury (Gehrke et al., 2018). IL-1R1 mediated microglial activation can impair cognition in humans (Guo et al., 2020). 

The objective of this study was to assess effect of *IL-1R1* KO on liver regeneration in aged mice. Twenty-four-month-old WT and *IL-1R1* KO mice were used to observe the recovery of liver after two-thirds PH.

## 2. Materials and methods

### 2.1. Animals 

*IL-1R1* KO and wild-type (WT) C57BL/6J mice (the ratio of the female to the male is 1:1) were purchased from Shanghai Laboratory Animals Inc. (Shanghai, China).* IL-1R1 *KO mice were obtained with a genetically disrupted *IL-1R1* gene as literature description (Glaccum et al., 1997)_ENREF_15. The mice were kept at the Experimental Animal Center of Henan Normal University. Feeding conditions were set at a temperature (24 ± 3 ˚C) and humidity (35 ± 5%) with 12 h day/night cycle; mice were free to get food and water. 

### 2.2. PH model

At 24 months old, the mice (weight from 20 to 30 g) were divided into 3 groups. Thirty-six *IL-1R1 *KO mice and 36 WT mice underwent 70% PH as previous description (Mitchell and Willenbring, 2008). Thirty-three WT mice underwent sham operation (SO), i.e. mice abdominal cavity was opened and liver lobes were ruffled but did not remove liver lobes, then the abdominal cavity was sutured. At 0, 2, 6, 12, 24, 30, 36, 72, 120, 168 and 192 h, 1% pentobarbital sodium (15 mL/kg, Beijing Huaye Huanyu Chemical Co., Ltd, Beijing, China) was injected into abdominal cavity to anesthetize the mice, then the mice were weighed. After bleeding from the inferior vena cava, the remnant liver weight was weighed. Liver/body weight were regarded as liver coefficient. The liver tissues were stored in the –80 ˚C. All animal experiments complied with the Animal Protection Law of China and animal ethics.

### 2.3. Quantitative real-time PCR assay

Total RNA in liver tissues were extracted by TRIzol reagent (Dingguo Biotechnology, Beijing, China) according to the instructions. The total RNA of 2 micrograms per tube was synthesized into cDNA by reverse transcription kit (Promega, Madison, WI, USA). Real-time quantitative PCR analysis of genes mRNA expressions were carried out using SYBR Green (Invitrogen, Carlsbad, CA, USA) reagent in Rotor-Gene 3000 PCR system (Corbett Robotics, Brisbane, Australia). *β-actin* expression was used as internal control. Genes expressions were quantified using 2–**∆∆Ct ** method (Livak and Schmittgen, 2001). The primer sequences are shown in Table.

**Table:** The genes primers sequence for qRT-PCR and their PCR annealing temperature.

Gene	Forward primer(5´- 3´)	Reverse primer(5´- 3´)	Annealing temperature
IL1R1	ACGATCGAAGCTGACCCAGGATCA	ACAAGGTCTGAGAACTGGCCCGT	57˚C
Fos	GTTTCAACGCCGACTACGAG	TTGGCACTAGAGACGGACAGA	59˚C
Jun	CAGAGTTGCACTGAGTGTGGC	GCAGTTGGTGAGAAAATGAAGAC	59˚C
Tnf-α	CGTCGTAGCAAACCACCAAGT	GGAGTAGACAAGGTACAACCCATC	58˚C
IL-6	CGTGGAAATGAGAAAAGAGTTGTG	CCAGTTTGGTAGCATCCATCAT	58˚C
Ifn-γ	TAGCCAAGACTGTGATTGCGG	AGACATCTCCTCCCATCAGCAG	58˚C
Mcp-1	TCAGCCAGATGCAGTTAACGC	TCTGGACCCATTCCTTCTTGG	58˚C
Ccr2	ATGCAAGTTCAGCTGCCTGC	ATGCCGTGGATGAACTGAGG	58˚C
Emr1	GGAAAGCACCATGTTAGCTGC	CCTCTGGCTGCCAAGTTAATG	58˚C
β-actin	CCGTAAAGACCTCTATGCCAACA	CGGACTCATCGTACTCCTGCT	58˚C

### 2.4. Western blotting analysis

The extracted total liver protein was analyzed using standard immunoblotting procedures. The densities of bands were quantified with the GE ImageQuant LAS 400 mini software. The antibodies used were: *cyclin D1*, PCNA, p-JNK1/JNK1, p-NF-κB1/NF-κB1, p-NF-κB2/NF-κB2, p-STAT3/STAT3, BAX, BCL2, active caspase-3, active caspase-8 and *β-actin* (Boaosen Biotechnology, Beijing, China).

### 2.5. Statistical analysis

Data are means ± SEM; differences between groups were statistically analyzed using the Student t-test by IBM SPSS 19.0 software (IBM Corp., Armonk, NY, USA). p < 0.05 was considered statistical significance.

## 3. Results

### 3.1. Decreased liver regeneration in IL-1R1 KO aged mice 

**Figure 1:**
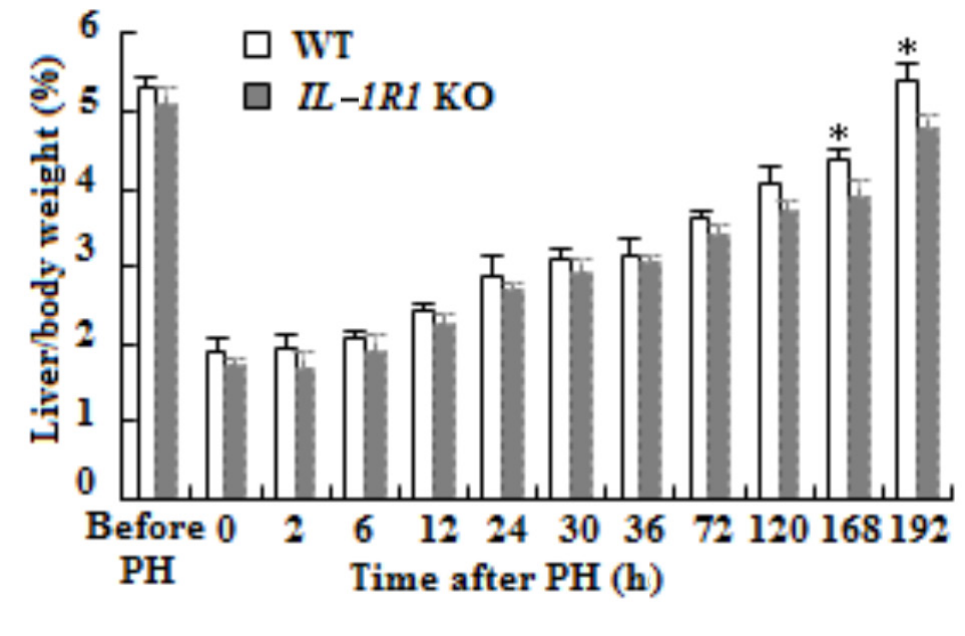
Liver weight recovery after PH. The liver weight/body weight ratio was demonstrated at different time points in WT and IL-1R1 KO mice after PH (n = 3, p < 0.05*).

After PH, liver coefficient increased during regeneration process, but difference was not obvious before 168 h; it was striking lower in *IL-1R1* KO mice than that of WT mice at 168 and 192 h (Figure 1, p = 0.039 and p = 0.027).

### 3.2. The mRNA expression levels of IL-1R1 gene 

**Figure 2:**
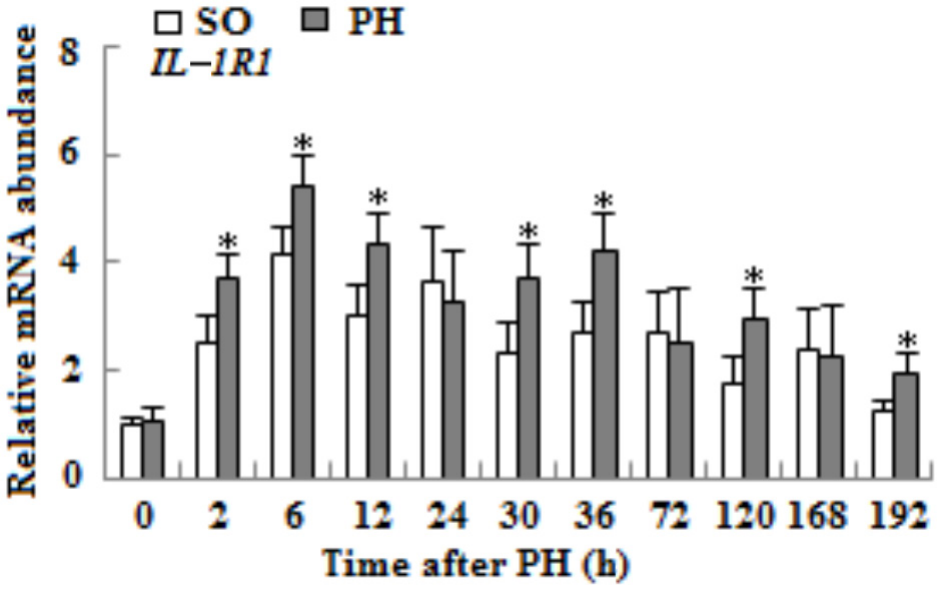
The mRNA expression of IL-1R1 gene in liver tissues of WT aged mice after SO or PH. The mRNA level of IL-1R1 was detected by qRT-PCR methods. β-actin mRNA was used to normalize gene expression (n = 3, p < 0.05*).

As shown in Figure 2, the mRNA expression of *IL-1R1* in remnant liver tissues of WT senescent mice significantly increased compared to SO groups at various time points, and the highest expression level was found at 6 h after PH (p < 0.05). 

### 3.3. The effect of IL-1R1 KO on the expressions of inflammation-related genes

**Figure 3:**
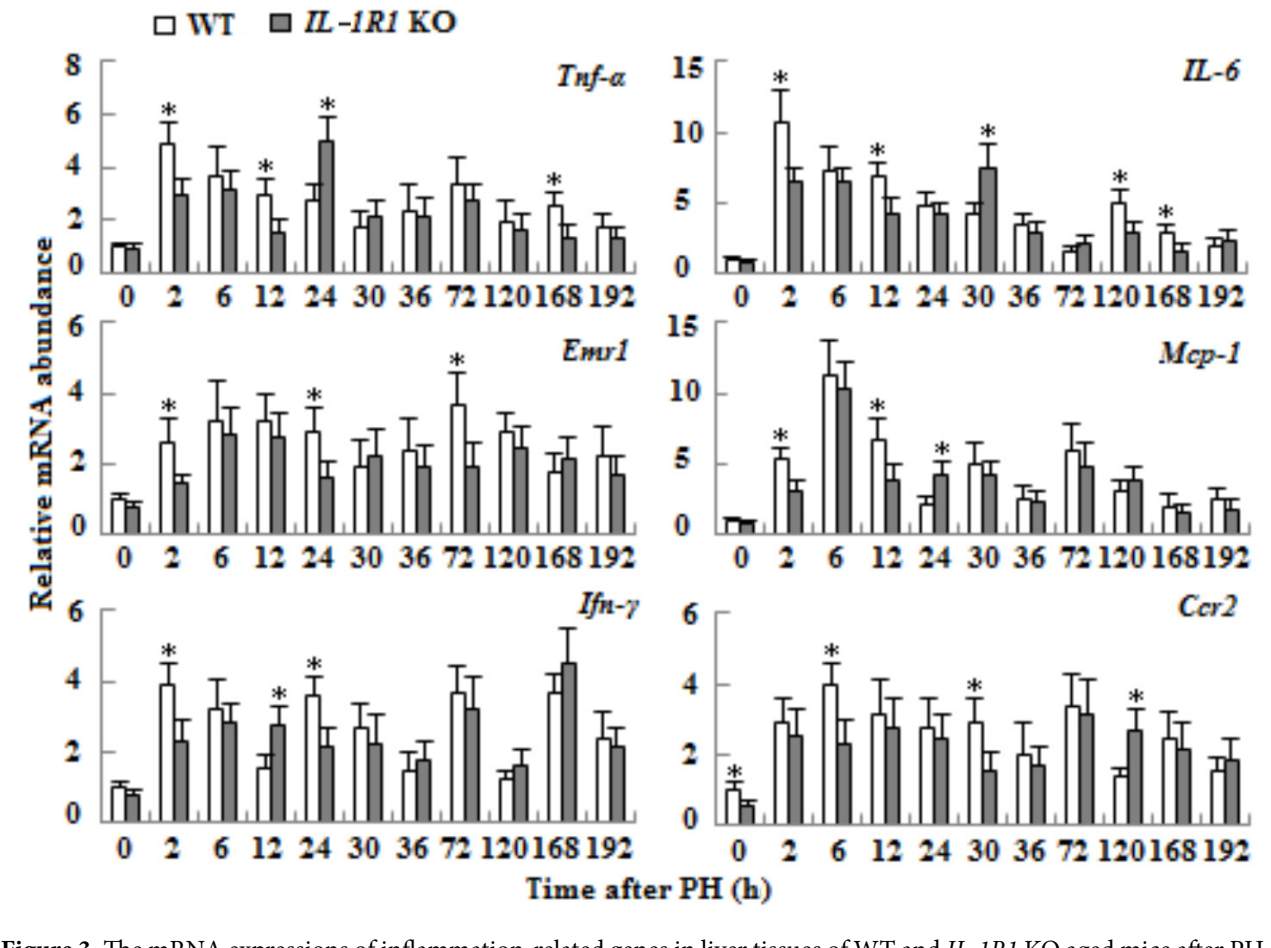
The mRNA expressions of inflammation-related genes in liver tissues of WT and IL-1R1 KO aged mice after PH. The mRNA levels of Tnf-α, IL-6, Ifn-γ, Mcp-1, Ccr2 and Emr1 were detected by qRT-PCR methods. β-actin mRNA was used to normalize gene expression (n = 3, p < 0.05*).

qRT-PCR method was used to detect the mRNA expressions of inflammation-related genes* Tnf-α*,* IL-6*, *Ccr2*, *Emr1*, *Ifn-γ* and *Mcp-1 *in regenerating liver tissues of aged mice after PH. The results showed that the mRNA expressions of these genes increased in the liver tissues of both types of aged mice. Compared with WT aged mice, the mRNA expressions of *Tnf-α*,* IL-6*, *Ccr2*, *Ifn-γ* and *Mcp-1* decreased and delayed; the mRNA expression of *Emr1 *declined to different extent in the liver tissues of* IL-1R1* KO aged mice (Figure 3, p < 0.05).

### 3.4. The mRNA expressions evaluation of immediate early genes 

**Figure 4:**
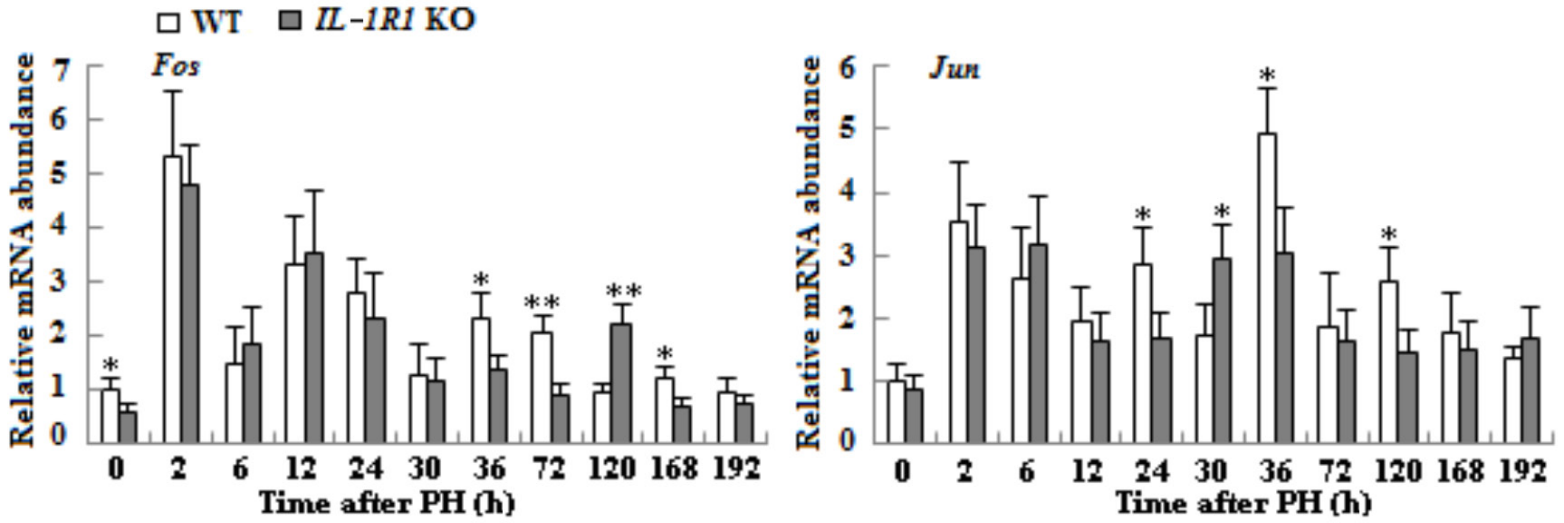
The mRNA transcription of the immediate early genes in liver tissues of both types of mice after PH. The mRNA expression of Fos and Jun was detected by qRT-PCR methods and β-actin mRNA was used as an internal control for normalization (n = 3, p < 0.05*, p < 0.01**).

The mRNA expressions of the immediate early genes *Fos *and* Jun *in remnant liver tissues of both types of aged mice increased during LR course. The expression elevation of *Fos* was extremely obvious at 2 h. Compared with WT mice, transcript level of *Fos* decreased and delayed from 36 to 168 h in *IL-1R1* KO aged mice; the expression of *Jun* declined and retarded from 24 to 120 h (Figure 4, p < 0.05 or p < 0.01). 

### 3.5. Delayed expressions of cyclins and proliferation marker in the liver tissues of IL-1R1 KO aged mice 

**Figure 5:**
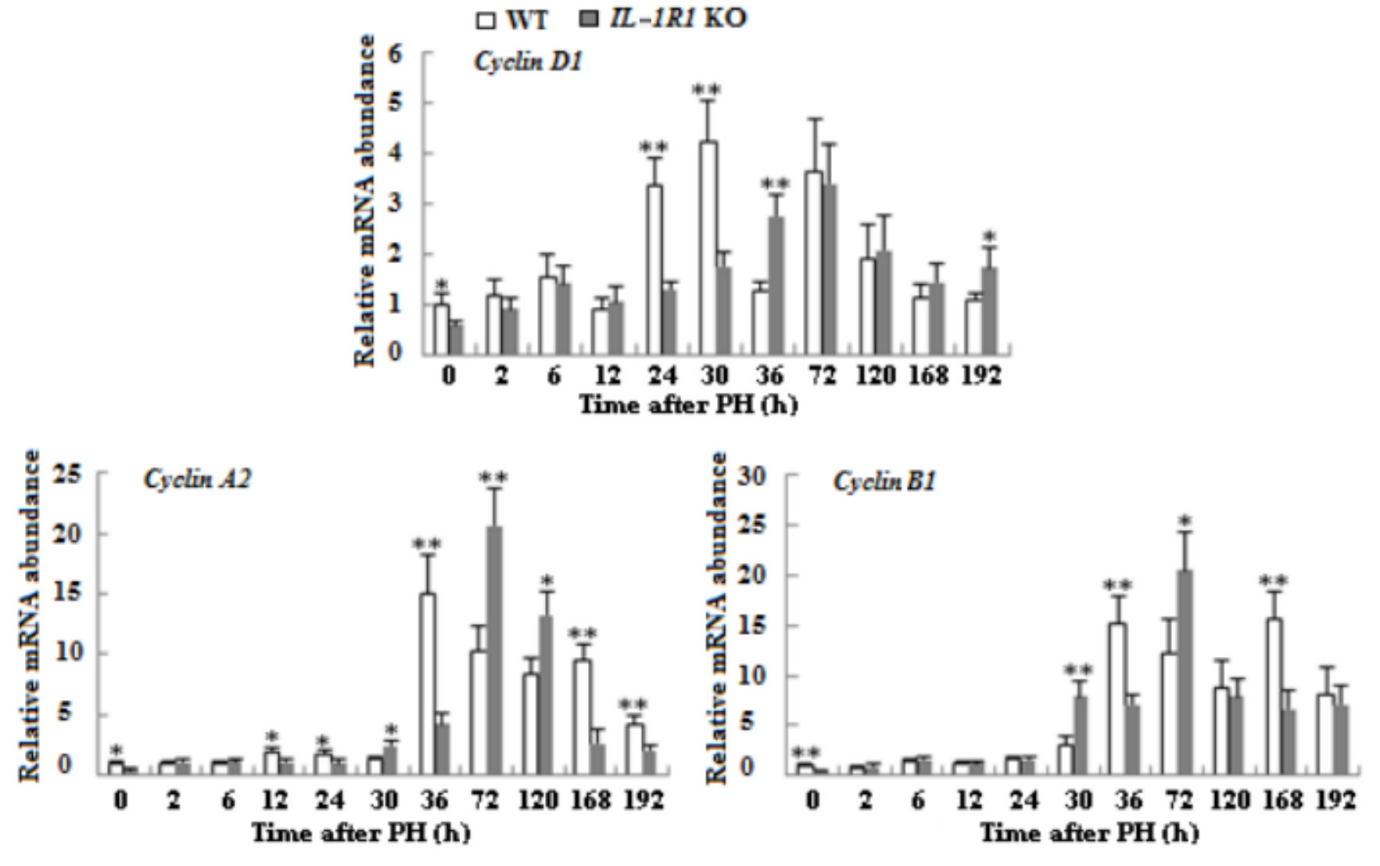
The mRNA expression of cyclin-associated genes in liver tissues of both types of mice after PH. The mRNA levels of cyclin D1, cyclin A2 and cyclin B1 were detected by qRT-PCR methods. β-actin mRNA was utilized to normalize gene expression (n = 3, p < 0.05*, p < 0.01**).

**Figure 6:**
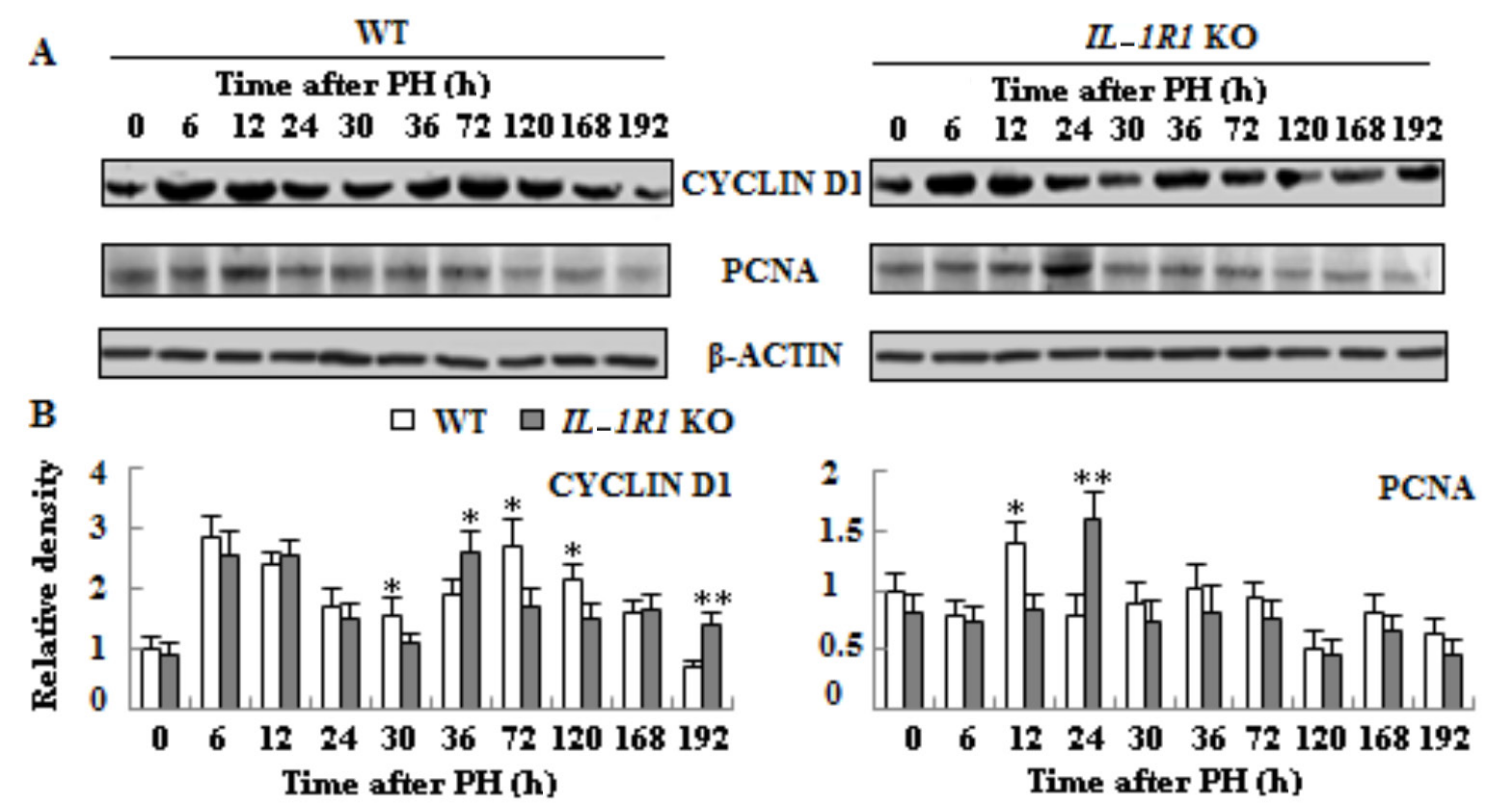
The expression levels of cyclin-related protein cyclin D1 and proliferation-related protein PCNA were detected by Western blotting methods in liver tissues of both type of mice after PH. β-actin was used as an internal control (n = 3, p < 0.05*, p < 0.01**).

To examine proliferation of hepatocytes after PH, we measured the mRNA expressions of cyclins by qRT-PCR technique and the protein expressions of *cyclin D1* and PCNA by Western blotting analysis. Compared to WT mice, the mRNA expressions of *cyclin D1*, *cyclin A2* and *cyclin B1* decreased and delayed in regenerating liver tissues of *IL-1R1* KO mice, and the mRNA transcription upregulation of *cyclin A2* and *cyclin B1* is extremely striking at middle and later phases of LR in both types of mice (Figure 5, p < 0.05 or p < 0.01). The protein expression of *cyclin D1* postponed at proliferation and termination phase of LR, and expression level of PCNA delayed at the initial stage of proliferation in the liver tissues of *IL-1R1* KO mice (Figure 6, p < 0.05 or p < 0.01).

### 3.6. The effects of IL-1R1 KO on the protein expressions of proliferation- and apoptosis-related genes in the liver tissues of two types of aged mice

**Figure 7:**
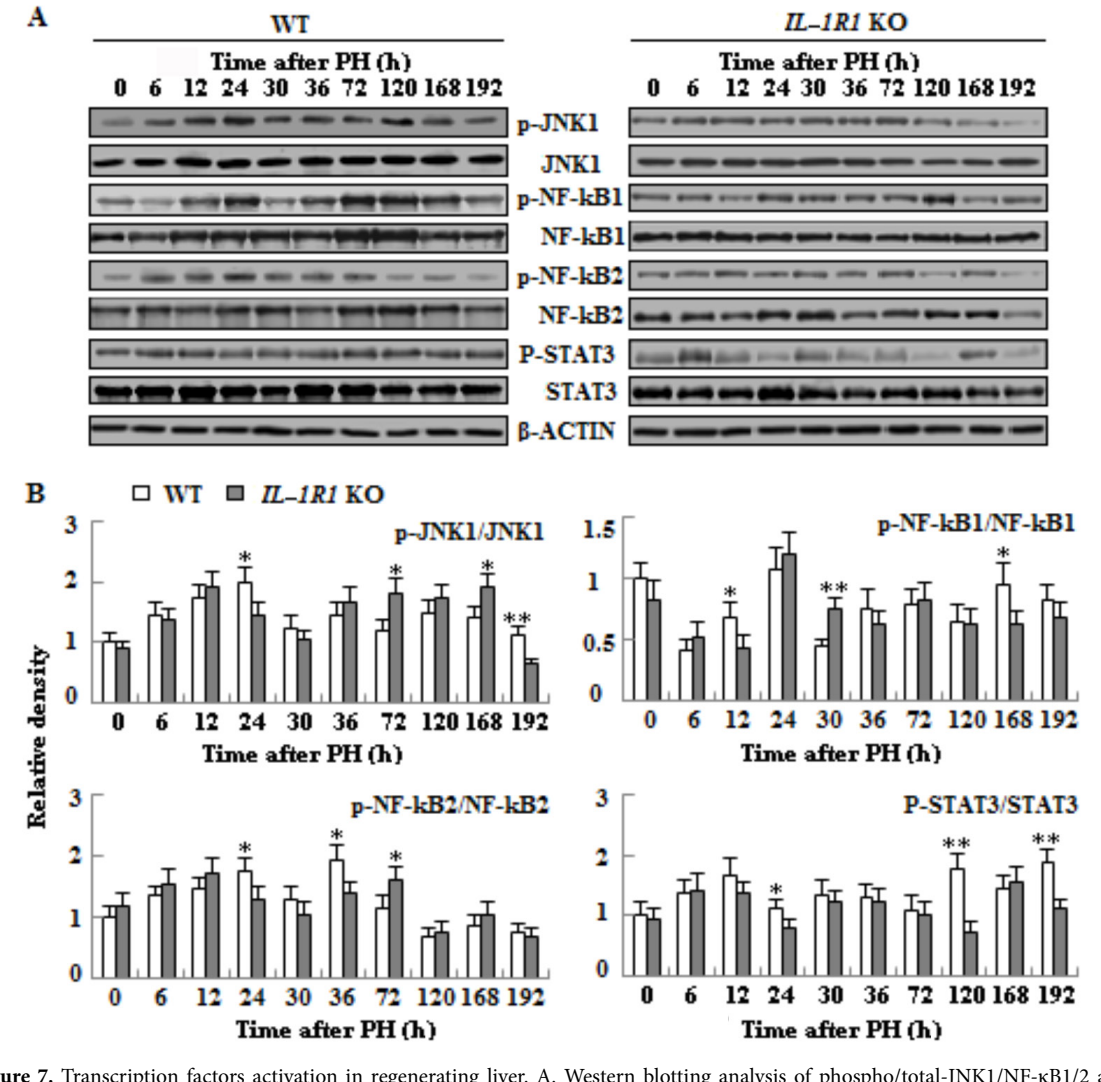
Transcription factors activation in regenerating liver. A. Western blotting analysis of phospho/total-JNK1/NF-κB1/2 and phospho/total-STAT3. B. Densitometric analysis of the results shown in A. β-actin was utilized as an internal control (n = 3, p < 0.05*, p < 0.01**).

**Figure 8:**
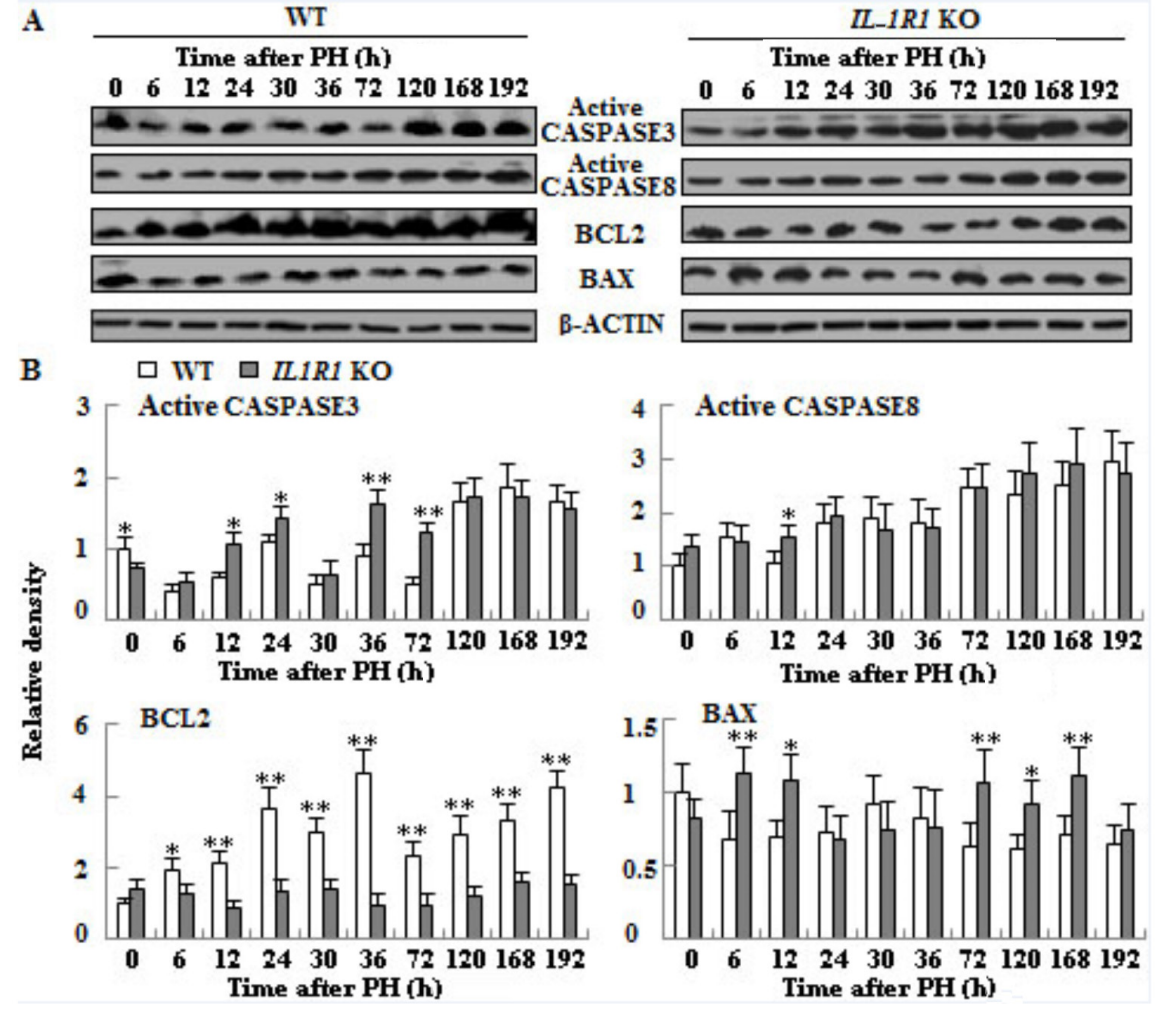
The proteins expressions of apoptosis-related genes in liver tissues of both types of aged mice after PH. A. Western blotting analysis of active caspase-3, active caspase-8, BCL2 and BAX proteins expressions. B. Densitometric analysis of the results shown in A. β-actin was used as an internal control (n = 3, p < 0.05*, p < 0.01**).

To discover the mechanism of impaired LR in *IL-1R1* KO aged mice, we detected the protein expressions of proliferation- and apoptosis-associated genes during regeneration process. Compared with WT mice, the phosphorylation of JNK1 was postponed to termination stage of LR in *IL-1R1* KO aged mice. The phosphorylation of NF-κB1 delayed to the 30 h and decreased again at 168 h of LR; the phosphorylation of NF-κB2 lagged to 72 h of LR. STAT3 phosphorylation decreased at proliferation and termination stage of LR (Figure 7, p < 0.05 or p < 0.01). The expression of proapoptotic executive protein active caspase-3 increased at proliferation phase; the expression of active caspase-8 elevated only at 12 h in the *IL-1R1* KO mice when compared to WT counterparts. The expression of apoptosis-inhibiting protein BCL2 had striking elevation during the whole regeneration process in the WT aged mice, however it only had tiny change in the *IL-1R1* KO mice. The expression of apoptosis-promoting protein BAX increased at 6–12 h and 72–168 h of LR in the *IL-1R1* KO aged mice when compared with WT aged mice (Figure 8, p < 0.05 or p < 0.01).

## 4. Discussion

Proinflammatory cytokine IL-1α or IL-1β is linked to IL-1R1 and can promote the expressions of various inflammatory genes including IL-1 itself (Alcaraz-Quiles et al., 2017). In the present study, the mRNA expression of *IL-1R1* significantly increased in regenerating liver tissues of WT aged mice after PH. Other liver injury studies have shown that the mRNA expression of *IL-1R1* in the pituitary, spleen and adrenal gland of young mice increased after lipopolysaccharide feeding (Pournajafi Nazarloo et al., 2003). The mRNA transcript of six inflammation-related genes was upregulated in regenerating liver tissues of both types of mice, but compared with WT control, their transcript decreased and delayed, which demonstrated inflammation attenuated in *IL-1R1* KO aged mice. The effect of *IL-1R1* KO on inflammation was consistent with previous description (Chen et al., 2007).

*Jun* and *Fos* as immediate early genes had important function in the initiation and proliferation stages of LR (Morello et al., 1990). Their mRNA expressions strikingly increased at the early phase of LR in regenerating liver tissues of both types of mice. Compared with WT aged mice, the transcription of *Fos *decreased and delayed at middle and later stages besides declined at 0 h; the transcription of *Jun *decreased and delayed at proliferation phase. The transcription upregulation of *Fos* and *Jun* can increase the expressions of genes related to cell cycle (Xiong et al., 1991). The reduction and lag of *Fos* and *Jun* mRNA expressions were similar to transcription changes of these cycle-associated genes; their expressions also decreased and delayed at proliferation and termination phases of LR. The protein expression of the proliferation marker PCNA delayed at proliferation phase of LR in *IL-1R1* KO aged mice when compared to WT mice, implying hepatocytes proliferation postponed in *IL-1R1* KO aged mice.

Cell proliferation and apoptosis are controlled by corresponding signaling pathways. IL-1 signaling has various effects, including angiogenesis and increased synthesis of acute phase response proteins by the liver (Dinarello, 1996). These responses are mediated by the activation of JNK protein and p38MAP kinase and upregulation of genes expressions stimulated by transcription factors NF-κB, C/EBPβ and AP-1 (O’Neill and Greene, 1998). JNK1 is very crucial for accelerated liver regeneration. Mice deficient in JNK1 can result in reduced LR after 2/3 PH (Seki et al., 2012). Inhibition of JNK1 can attenuate mouse livers regeneration after portal vein ligation for staged hepatectomy (Langiewicz et al., 2018). The role of JNK2 is elusive. Sabapathy et al. (2004) indicated that the loss of JNK2 contribute to cell proliferation; other study, however, demonstrated JNK2 seems dispensable (Schaefer et al., 2015) or no role in LR (Das et al., 2011). In our experiment, the delayed JNK1 phosphorylation perhaps results in reduced LR at the last two time points in *IL-1R1* KO mice. IL1R1 combines with IL-1 on the cell surface which can upregulate inflammation and affect NF-κB signaling (Rhodes et al., 2015). NF-κB plays a key role in maintaining liver homeostasis by regulating the transcription of genes (Majidinia et al., 2017). NF-κB can regulate the expression of *cyclin D1* (Guttridge et al., 1999) and has antiapoptotic functions (Luedde and Schwabe, 2011). Compared with WT mice, the decreased and delayed NF-κB phosphorylation perhaps led to the delayed expression of *cyclin D1* and increased apoptosis; increased apoptosis led to less cell proliferation in the liver of *IL-1R1* KO mice. The increase of liver cell number is mainly via the IL-6/STAT3 pathway (Fujiyoshi and Ozaki, 2011). STAT3 is induced during liver regeneration mainly dependent on IL-6 (Cressman et al., 1996). Attenuation of IL-6 signaling may lead to the decreased phosphorylation of STAT3 at mid and late stages in the livers of *IL-1R1* KO mice. STAT3 was necessary for the activation of *cyclin D1* (Li et al., 2002), perhaps besides NF-κB the decreased phosphorylation of STAT3 also caused the delayed expression of *cyclin D1*, which delayed the proliferation of liver cells in *IL-1R1* KO mice, as demonstrated by PCNA. IL-6/STAT3 pathways can upregulate the expression of antiapoptosis protein BCL-2 to protect against cell death (Fujiyoshi and Ozaki, 2011). As shown in Figure 8, the expression upregulation of BCL-2 protein is especially remarkable from 6 to 192 h, and the expression of proapoptotic protein Bax did not increase in regenerating liver tissues of WT aged mice when compared to* IL-1R1* KO mice; which may lead to less activation of caspase-3 in the liver of WT mice, therefore there are more cell proliferation in WT miceThus decreased expression of *IL-6* and STAT3 probably caused impaired LR at 168 and 192 h in *IL-1R1* KO mice. We found the mRNA expression of *Fos* obviously declined at 168 h, and the mRNA expression decrease of *cyclin A2* is extremely striking at 168 and 192 h in the liver tissues of *IL-1R1* KO mice, perhaps *Fos *and* cyclin A2* play a more important role at this phase of LR. 

Inflammation seems like a double-edged sword. On the one hand, it is essential for the defense of the main body itself; on the other hand, if the body fails to prevent the inflammatory reaction, it will destroy the cells and tissues and lead to the occurrence of chronic immune-mediated inflammatory diseases, allergies, or cancer. Response to different stress states, hepatic inflammation protects liver cells from injury, repairs tissues damage and promotes homeostasis. Several cellular components, including the dual function IL-1α, are released during liver injury, which induced aseptic inflammation and tissues repair (Brenner et al., 2013). The margin between benefit and damage is very narrow (Dinarello, 1997). Previous reports suggested that inflammation in liver injury caused by chemical toxic is harmful (Yu et al., 2014; Gehrke et al., 2018), but inflammation plays a beneficial role in PH model; Yin et al. (2011) found that higher inflammatory response can enhance LR; Furuya et al. (2013) reported that decreased proinflammatory cytokines impaired LR after 2/3 PH. Tan et al. (2016) demonstrated the complexity of IL-1R1 pathway: compared with WT control group after 1/3 PH, LR of *IL-1R1* KO young mice decreased at the early stages (24 h), but increased at the later stages. Increased inflammatory signaling was often observed during aging; inflammation in the liver potentially mediates age-related changes (Franceschi et al., 2000; Gee et al., 2005). This study showed that* IL-1R1* KO reduced and delayed the mRNA expressions of inflammation-related genes to different extents, which caused impaired LR in aged mice at later stage (168–192 h). Perhaps proper inflammation helps to regeneration after liver partly surgical excision in the elderly. 

## References

[ref1] (2017). Polymorphisms in the IL-1 gene cluster influence systemic inflammation in patients at risk for acute-on-chronic liver failure. Hepatology.

[ref2] (2013). The interleukin-1 receptor family. Seminars in Immunology.

[ref3] (2013). Decoding cell death signals in liver inflammation. Journal of Hepatology.

[ref4] (2007). Identification of a key pathway required for the sterile inflammatory response triggered by dying cells. Nature Medicine.

[ref5] (1996). Liver failure and defective hepatocyte regeneration in interleukin-6-deficient mice. Science.

[ref6] (2011). The role of JNK in the development of hepatocellular carcinoma. Genes & Development.

[ref7] (1996). Biologic basis for interleukin-1 in disease. Blood.

[ref8] (1997). Induction of interleukin-1 and interleukin-1 receptor antagonist. Seminars in Oncology 24 (3 Suppl.

[ref9] (2006). Liver regeneration. Hepatology.

[ref10] (2019). IL1R1 is required for celastrol’s leptin-sensitization and antiobesity effects. Nature Medicine.

[ref11] (2000). Inflamm-aging. An evolutionary perspective on immunosenescence. Annals of the New York Academy of Sciences.

[ref12] (2011). Molecular mechanisms of liver regeneration and protection for treatment of liver dysfunction and diseases. Journal of Hepato-Biliary-Pancreatic Sciences.

[ref13] (2013). Interleukin-17A plays a pivotal role after partial hepatectomy in mice. Journal of Surgical Research.

[ref14] (2005). Modulation of apolipoprotein E and interleukin-1beta in the aging liver. Experimental Gerontology.

[ref15] (2018). Hepatocyte-specific deletion of IL1-RI attenuates liver injury by blocking IL-1 driven autoinflammation. Journal of Hepatology.

[ref16] (1997). Phenotypic and functional characterization of mice that lack the type I receptor for IL-1. Journal of Immunology.

[ref17] (2009). Interleukin-1 (IL-1): a central regulator of stress responses. Front Neuroendocrinology.

[ref18] (2017). IL-1 family cytokines use distinct molecular mechanisms to signal through their shared co-receptor. Immunity.

[ref19] (2020). Visceral adipose NLRP3 impairs cognition in obesity via IL-1R1 on CX3CR1+ cells. Journal of Clinical Investigation.

[ref20] (1999). NF-κB controls cell growth and differentiation through transcriptional regulation of cyclin D1. Molecular and Cellular Biology.

[ref21] (2003). Aging reduces proliferative capacities of liver by switching pathways of C/EBPalpha growth arrest. Cell.

[ref22] (2019). Augmenter of liver regeneration: Essential for growth and beyond. Cytokine & Growth Factor Reviews.

[ref23] (2018). JNK1 induces hedgehog signaling from stellate cells to accelerate liver regeneration in mice. Journal of Hepatology.

[ref24] (2001). Analysis of relative gene expression data using real-time quantitative PCR and the 2(-. Methods.

[ref25] (2002). STAT3 contributes to the mitogenic response of hepatocytes during liver regeneration. Journal of Biological Chemistry.

[ref26] (2011). NF-kappaB in the liver--linking injury, fibrosis and hepatocellular carcinoma. Nature Reviews Gastroenterology & Hepatology.

[ref27] (2017). Co-inhibition of notch and NF-kappaB signaling pathway decreases proliferation through downregulating IkappaB-alpha and Hes-1 expression in human ovarian cancer OVCAR-3 cells.

[ref28] (2008). A reproducible and well-tolerated method for 2/3 partial hepatectomy in mice. Nature Protocols.

[ref29] (2020). β-glucan induces protective trained immunity against mycobacterium tuberculosis infection: a key role for IL-1. Cell Reports.

[ref30] (1990). Differential regulation and expression of jun, c-fos and c-myc proto-oncogenes during mouse liver regeneration and after inhibition of protein synthesis. Oncogene.

[ref31] (1998). Signal transduction pathways activated by the IL-1 receptor family: ancient signaling machinery in mammals, insects, and plants. Journal of Leukocyte Biology.

[ref32] (2003). Modulation of type I IL-1 receptor and IL-1 beta mRNA expression followed by endotoxin treatment in the corticotropin-releasing hormone-deficient mouse. Journal of Neuroimmunology.

[ref33] (2015). Computational modelling of NF-kappaB activation by IL-1RI and its co-receptor TILRR, predicts a role for cytoskeletal sequestration of IκBα in inflammatory signalling. PLoS One.

[ref34] (2004). Distinct roles for JNK1 and JNK2 in regulating JNK activity and c-Jun-dependent cell proliferation. Molecular Cell.

[ref35] (2019). Cathepsin L-deficiency enhances liver regeneration after partial hepatectomy. Life Sciences.

[ref36] (2015). Bone marrow-derived c-jun N-terminal kinase-1 (JNK1) mediates liver regeneration. Biochimica et Biophysica Acta (BBA)-Molecular Basis of Disease.

[ref37] (2012). A liver full of JNK: signaling in regulation of cell function and disease pathogenesis, and clinical approaches. Gastroenterology.

[ref38] (2011). Proximal gut mucosal epithelial homeostasis in aged IL-1 type I receptor knockout mice after starvation. Journal of Surgical Research.

[ref39] (2016). The role of IL-1 family members and Kupffer cells in liver regeneration. BioMed Research International.

[ref40] (1991). Human D-type cyclin. Cell.

[ref41] (2011). Enhanced liver regeneration in IL-10–deficient mice after partial hepatectomy via stimulating inflammatory response and activating hepatocyte STAT3. The American Journal of Pathology.

[ref42] (2014). Protective effects of the total saponins from Dioscorea nipponica Makino against carbon tetrachloride-induced liver injury in mice through suppression of apoptosis and inflammation. International Immunopharmacology.

